# Detection of Herpesviruses (Predominantly HHV-6) in Patients with Guillain–Barré Syndrome

**DOI:** 10.3390/biomedicines13040845

**Published:** 2025-04-01

**Authors:** Jéssica Gonçalves Pereira, Tainá Madeira Barros Pontes, Fernanda Martins Maia Carvalho, André Borges Ferreira Gomes, Rafael Santos Erbisti, Ivanildo Pedro de Sousa Junior, Jeová Keny Baima Colares, Danielle Malta Lima, Vanessa Salete de Paula

**Affiliations:** 1 Laboratory of Molecular Virology and Parasitology, Oswaldo Cruz Institute, Fiocruz, Rio de Janeiro 21040-360, Brazil; jessica-gpereira@hotmail.com (J.G.P.); ivanildo.sousa@ioc.fiocruz.br (I.P.d.S.J.); 2 Graduate Program in Medical Sciences, University of Fortaleza, Fortaleza 60811-905, Brazil; tainapontes@hotmail.com (T.M.B.P.); fernandamaia@unifor.br (F.M.M.C.); andrebfg@gmail.com (A.B.F.G.); kenycolares@unifor.br (J.K.B.C.); 3 Neurology Department, Fortaleza General Hospital, Fortaleza 60150-160, Brazil; 4 Department of Statistics, Institute of Mathematics and Statistics, Fluminense Federal University, Niterói 24210-240, Brazil; rerbisti@id.uff.br

**Keywords:** HHV-6, Guillain–Barré syndrome, herpesvirus, neuroinfections

## Abstract

**Background/Objectives:** Guillain–Barré syndrome (GBS) is a neurological disease that affects the peripheral nerves. The exact cause of this condition is still uncertain, but cross-reactivity between pathogen antigens and nervous tissue may play a crucial role in disease pathogenesis. *Roseolovirus humanbeta6* (HHV-6), a neurotropic virus with latency capacity, may be considered a significant candidate for triggering or worsening neurological conditions. In this study, we aimed to investigate the detection of HHV-6 in the CNS from GBS patients. Of the 23 individuals suspected of having GBS, 13 were confirmed as having the disease. We then analyzed the frequency of herpesviruses in the cerebrospinal fluid (CSF) samples from these 13 individuals with GBS who were also tested for enteroviruses and arboviruses and had negative results. **Results:** After extraction of viral DNA from CSF samples, real-time PCR (qPCR) methodology was used to analyze the frequency and viral load of herpesviruses. Sociodemographic and clinical data were collected for analysis and verification through statistical tests such as Fisher’s exact test and the Mann–Whitney test. Thirteen individuals diagnosed with GBS were tested. Among the 13 patients analyzed, 61.5% were men, 38.4% (5/13) tested positive for HHV-6, 61.5% of the patients tested positive for a herpesvirus, 30.8% had two viral DNAs identified, and one patient presented three different strains. Patients who tested positive for HHV-6 had a significantly longer average length of stay (25.6 days versus 11 days for negative patients). HHV-6 was the most frequent subtype detected in patients positive for herpesviruses (62.5%, 5/8). **Discussion/Conclusions:** Our results show a possible relationship between HHV-6 and GBS cases despite the small number of patients, raising the question of whether the presence of HHV-6 influences GBS, since its investigation using qPCR is not routinely used. This may have some impact on prognosis, since antiviral therapy is not included in the standard treatment of GBS patients, and viral DNA load may interfere with the inflammatory process of GBS.

## 1. Introduction

Guillain–Barré syndrome (GBS) is the major cause of acute flaccid paralysis, mostly due to demyelination of peripheral nerves, developing its symptoms over several days to weeks [1]. The first description using clinical and laboratory criteria was published in 1916 by Georges Guillain, Jean-Alexandre Barré, and Andre Strohl [2]. GBS is commonly characterized by numbness, progressive ascending weakness, paralysis, tingling, pain, and autonomic dysfunction [3]. Similar to other diseases, both genetic and environmental factors play important roles in triggering GBS [4]. The global incidence of GBS is estimated to range between 1.1/100,000 and 1.8/100,000 individuals per year and increases with age, with 100,000 new cases reported annually worldwide [5].

GBS can be considered a post-infectious autoimmune disease (AD) [6]. Infections such as *M. pneumoniae*, *Haemophilus influenza*, HCMV, HSV, EBV, hepatitis E virus, and *C. jejuni* have been reported to be associated with the development of GBS [7]. Although it is clearly associated with molecular mimicry [6], acute motor axonal neuropathy (AMAN), which targets axon membranes, is the only phenotype that has been confirmed to fulfill all four criteria of molecular mimicry [8]. There is also acute inflammatory demyelinating polyneuropathy, which is typically associated with immune injury to the myelin sheath, which has not yet been related. Although these criteria have existed for some years, they are challenging for several reasons, such as uncertainty about the timing of infection, the fact that not all individuals develop AD despite being infected, latency characteristics, lack of sufficient epidemiological coverage, limitations of genetic studies in humans, and the absence of compatible murine models [9]. So that the etiology of ADs does not remain enigmatic, it is of utmost importance to conduct further studies in groups of individuals who may present this relationship, as described in the manuscript.

Studies addressing GBS are important and necessary, as in addition to the symptoms mentioned above, this disease can trigger severe respiratory failure in 20–30% of patients [2,5]. Despite existing treatments, such as intravenous immunoglobulin therapy (IVIG) and plasmapheresis, approximately 20% of patients develop severe disabilities and lifelong sequelae. Furthermore, new episodes can develop, and the GBS recurrence rate is estimated at 1–6% [5].

Human herpesvirus 6 (HHV-6) is divided into two very similar species of beta-herpesviruses: HHV-6A and HHV-6B [10]. Although these viruses have more than 95% genomic homology and both associated virions are structurally composed of a nucleus containing double-stranded linear DNA protected by an icosahedral capsid, they present with distinct epidemiological and biological characteristics and clinical behaviors [11]. HHV-6A, first isolated by Salahuddin et al. in patients with lymphoproliferative diseases or acquired immunodeficiency syndrome, is known to have a greater virulence capacity and cytolytic character, whereas HHV-6B, isolated by Yamanishi et al. in lymphocytes from patients with a sudden rash, is known as the agent responsible for the sudden rash present in Roseola infantum, a disease characterized by high fever and skin rash that presents in childhood [12].

Similar to other human herpesviruses, HHV-6 infection is common, with a seroprevalence of close to 90% (antibody detection) [11]. Owing to its latent characteristics, the salivary glands usually function as the main reservoir of HHV-6, meaning that this virus is generally transmitted through saliva [13]. Following contact with the host, HHV-6 infects diverse human cells; however, it has a greater affinity for CD4+ T lymphocytes [10].

HHV-6 is known to infect cells of the central nervous system (CNS) and cause neurological manifestations [14]. Current studies have focused on neurotrophic properties, investigating possible links to encephalitis, seizure episodes, Alzheimer’s disease (AD), Guillain–Barré syndrome (GBS), and multiple sclerosis (MS) [11,12]. Active infection or latent stage can influence the balance between immune and glial cells in the CNS, and although the latter of these stages is not responsible for triggering the aforementioned diseases, it can worsen the patient’s inflammatory state, influencing disease progression. Furthermore, HHV-6 has a high affinity for CD4+ T lymphocytes, which may facilitate the entry of the virus into the CNS via the blood–brain barrier (BBB) [15].

The high similarity between HHV-6A and HHV-6B makes it difficult to investigate these viruses separately [11]. Although both are neurotropic and manifest in the CNS, studies have indicated that HHV-6A has a greater neurovirulence [11]. However, recent studies have shown that primary infection or reactivation in immunocompromised HHV-6B individuals poses an exceptionally high risk of encephalitis and associated encephalopathies, which are common, permanent, and debilitating conditions [10].

Despite all the advances made in understanding viral triggers and GBS, there is still a high proportion of cases that remain without identified triggers. There is a lack of current studies on herpesviruses, particularly HHV-6, regarding their relationship with GBS, which intensifies the need for further investigation. The aim of this study was to evaluate the frequency the Herpesviridae family members in patients with GBS and to determine whether their presence, linked to a high viral load, may influence the severity of GBS symptoms.

## 2. Materials and Methods

### 2.1. Study Design

This is a retrospective analysis of biological samples from participants in a longitudinal and prospective study conducted in a tertiary care hospital for neurological diseases, located in a city with approximately 2.5 million inhabitants in northeast Brazil. Samples were collected from April 2016 to March 2017 and stored in a biorepository. An analysis of the viral DNA of herpesviruses was carried out in the first half of 2024 in the Virology and Molecular Parasitology Laboratory at FIOCRUZ/Rio de Janeiro.

### 2.2. Study Participants

The patient cohort included individuals aged over 18 years with a confirmed diagnosis of Guillain–Barré syndrome (GBS) based on the Brighton criteria [16,17]. Participants with serum potassium levels below 3.0 mmol/L or cerebrospinal fluid (CSF) pleocytosis exceeding 50 cells/mm^3^ were excluded [18].

### 2.3. Sample Collection and Processing

Data were collected during hospitalization through structured interviews, analysis of medical records, and sample collection. CSF samples were obtained according to routine hospital admission policies. The samples were transported in styrofoam thermal boxes containing recyclable ice bags and stored until arrival at the laboratory. Biological material was maintained at −80 °C until use.

### 2.4. Extraction of DNA

Viral DNA was extracted from the CSF samples. Cell-free DNA was isolated with a QIAamp DNA Mini Kit (QIAGEN, Hilden, Germany) using the DNA spin column technique, from 200 μL of sample, according to the manufacturer’s instructions. DNA was finally eluted in 50 μL of nuclease-free water in order to concentrate it. Two positive and two negative controls (water-free) were used for each set of 10 samples. The final samples were stored at −80 °C until processing.

### 2.5. Detection and Quantification of Viral Load Using qPCR

Detection and quantification of the viral loads of HSV-1, HSV-2, VZV, EBV, HCMV, HHV-6, HHV-7, and HHV-8 in the CSF samples were performed using real-time PCR (qPCR) according to the protocol published by Carneiro et al., 2022 [19]. Oligonucleotides and probes target highly conserved regions of the viral genome. As previously reported by Sousa et al., viral RNA extracted from CSF samples was subjected to broad-reactive real-time RT-PCR (rRT-PCR) of enteroviruses [20].

### 2.6. Statistical Analysis

The patients’ data were separated into 2 groups for statistical analysis: HHV-6 positive and HHV-6 negative patients. Fisher’s exact test [21] was applied to analyze the association between patient characteristics. Differences in CSF parameters and admission strength between the groups were assessed using the Mann–Whitney test [22]. The same test, based on paired samples, was employed to compare viral loads in patients presenting more than one viral DNA to evaluate the viral load of HHV-6 and other viruses. Statistical significance was set at 5%. Statistical analyses were conducted using R software version 4.2.2 (a programming language for performing statistical analysis using collected data [23]).

## 3. Results

### 3.1. Sociodemographic Data, Clinical Manifestations of GBS Patients, and Their Respective Medians

During the study period, 23 patients with suspected GBS were evaluated, but the diagnosis of GBS was confirmed in 13 patients based on neurological findings from CSF and electroneuromyography. Eight patients were male (61.5%) and had a median age of 50 years (interquartile range, IQR 38.5–68.5). Systemic symptoms were reported in 10 patients (77%), the most prevalent being arthralgia (*n* = 4, 31%), followed by fever (*n* = 3, 23%) and myalgia (*n* = 3, 23%). The median time to onset of neurological symptoms was eight days (IQR 5.0–17.5). The most prevalent initial neurological symptoms were paresthesia (*n* = 10, 77%) and paresis (*n* = 6, 46%) in the upper and lower limbs. Only two cases (16%) presented with encephalitic symptoms. The median length of hospital stay was 12 days (IQR 7.0–17.0). The most common electromyoneurographic alteration was the presence of a demyelination pattern with secondary axonal damage (6.46%). There was a higher prevalence of the variant form of acute inflammatory demyelinating polyradiculoneuropathy (9.69%).

Regarding HHV-6, five individuals were positive (38.4%), all of whom were co-infected with another herpesvirus. HHV-6-positive patients had a mean age of 57.8 years, while HHV-6-negative individuals were approximately 50.2 years old (*p*-value 0.130). The majority of HHV-6-positive patients were male (80% (4/5) (*p*-value 0.565). The mean duration of hospital stay was 25.6 and 11 days for positive and negative individuals, respectively (*p*-value 0.495). On the first day of symptoms, HHV-6-positive individuals presented with symptoms such as muscle weakness (paresis, 80%), tingling/numbness (paresthesia, 40%), paraplegia (20%), and hypotension (20%), whereas symptoms in negative individuals included paresis and paresthesia (25% and 100%, respectively) (*p*-value 0.146). The distribution of systemic symptoms was balanced in both groups, and only one HHV-6-positive individual presented with somnolence (20%, 1/5) (*p*-value 0.954). Regarding CSF, the mean glucose level in HHV-6-positive patients showed a tendency to be lower than that in HHV-6-negative patients. All HHV-6-positive individuals developed progressive tetraparesis. Regarding the electroneuromyography investigation, both groups presented axonal damage, demyelination, or demyelination with secondary axonal damage. The final electrophysiological diagnosis in the HHV-6-positive individuals was AIDP in 60% (3/5) and AMSAN in 40% (2/5). No differences were found in sociodemographic, clinical, or laboratory variables between the groups (Table 1).

### 3.2. CSF Characteristics and Clinical Manifestations of GBS Patients for Admission

The median interval between the onset of neurological symptoms and cerebrospinal fluid collection was nine days (IQR 6.3–17.3). CSF investigation showed similar values in both the HHV-6-positive and HHV-6-negative groups, with a cell count of 1 cell/mm^3^ (IQR 1.0–3.0), total protein of 92 mg/dL (IQR 37.5–115.5), and glucose level of 60 mg/dL (IQR 52.0–69.3). No statistical difference was observed (Table 2).

### 3.3. Frequency of Herpesviruses and Viral Load in Samples from GBS Patients

Of the 13 patients enrolled, 61.5% (8/13) tested positive for a member of the Herpesviridae family. Among the positive cases, four patients harbored DNA from two viruses, and one patient was infected with three different viral genomes (HHV-6, HSV-2, and HCMV). The highest viral loads were observed for HHV-6, which was also the most prevalent, detected in 62.5% (5/8) of the cases (Table 3).

## 4. Discussion

Our findings revealed a high frequency of herpesviruses in this group of patients, particularly HHV-6, which has not been previously described in the literature. In our study, out of a total of 13 individuals diagnosed with GBS, we focused on CSF samples, whereas most studies usually use blood/plasma samples. Here, HHV-6 was detected in CSF using qPCR, indicating a lytic infection. The results may suggest active replication and a link to the CNS. They show a high viral load of HHV-6, including co-infection with other herpes types.

The herpesvirus genome was identified in 62% of the CSF samples, with positive detection of HHV-6 (38%), HSV-2 (23%), HCMV (23%), HSV-2 (8%), VZV (8%), EBV (8%), and HHV-8 (8%). A previous study involving 30 participants conducted in the Neurology Department at El Hadara University Hospital to evaluate the etiological correlation between CMV, EBV, and HHV-6 infection and GBS found positivity rates of 10% for HHV-6, 26.7% for HCMV, and 30% for EBV [24]. These results highlight the need to investigate the presence of other viruses in patients with GBS using more precise techniques for viral detection. The average age of HHV-6-positive individuals in our study, representing 38.4% (5/13) of the sample, was 57.8 ± 22.88 years. These age results are comparable to those of studies conducted in Brazil, the United States of America, China, Mexico, Spain, Korea, Japan, the European Union, Australia, Ireland, and Canada [24,25]. Males represented 61.5% (8/13) of the sample, which is consistent with the literature, where previous reports also showed a higher prevalence of males affected by this syndrome [16,26].

Other herpesviruses, such as HCMV and VZV, found in patients with GBS were detected in the present study. Herpesvirus reactivation is commonly triggered after infection, with *Campylobacter jejuni* enteritis and cytomegalovirus (HCMV) infection being the most common [27]. In this study, HCMV presented a frequency of 23%, while a prior study conducted in the northeastern region of Brazil in 2020 reported a frequency of 15.3% [27]. The HCMV-positive patients in our study also experienced a more severe disease course, longer hospital stays, and more intense sensory damage than those in this latter study. Varicella-zoster virus (VZV), which has the potential for immune-mediated demyelination, is also notable among patients with GBS [28,29]. Particularly, in cases of Herpes Zoster, a condition affecting approximately 1 million Americans per year, GBS triggered by this infectious episode shows a more severe response, mainly affecting younger individuals (18–64 years old) [28]. In our study, VZV presented with a frequency of 7.5%, while codetection with HHV-6 was observed. Islam et al. reported a group of patients with sociodemographic data similar to ours but with a lower frequency of VZV (1.3%) [29]. Their analysis of the CSF supports the notion that detecting viral DNA in the CSF may indicate direct CNS involvement, underscoring the importance of studying the presence of herpesviruses in patients with neurological conditions [29,30].

Among HHV-6-positive individuals, 80% (4/5) were women, and the median length of hospital stay for positive patients was 25.6 days compared with 11 for HHV-6-negative patients. According to studies by Kim et al. and Leeuwen et al., the median length of hospital stays for GBS patients ranged from 10 to 38 days, depending on the severity of the symptoms [31,32]. Our data indicated that patients with longer hospital stays and greater disability also tested positive for HHV-6. On the first day after symptom onset, HHV-6-positive patients presented with muscle weakness (paresis, 80%), tingling/numbness (paresthesia, 40%), paraplegia (20%), and hypotension (20%). In contrast, 25% and 100% of the negative individuals presented symptoms of paresis and paresthesia, respectively.

These results indicate the possibility that HHV-6 may influence a more severe symptomatology in patients due to its replication process along with the development of GBS, showing a high prevalence of motor deficits as presenting symptoms and hypotension, also indicating autonomic involvement.

CSF analysis remains one of the most important parameters for the diagnosis of GBS, regardless of the trigger [33]. CSF analysis in our study showed a profile compatible with GBS, characterized by high protein content due to cytological dissociation of albumin, resulting from an inflammatory response [33]. There were no differences in CSF parameters between the groups.

HHV-6 had the highest viral load and was most frequently detected in the patient groups (62.5%). Hypotension, upper and lower limb paresis, muscle pain, and paraplegia were the most frequently reported clinical manifestations by HHV-6-positive patients with GBS in this study, and all of them also developed progressive tetraparesis. Electroneuromyography revealed that 60% (3/5) of the patients presented with AIDP, but no significant differences were observed between the groups. The fact that no differences were observed in CSF or EMG patterns between the groups makes qPCR analysis for HHV-6 detection even more important as a biomarker in the detection of patients with a possibly worse prognosis. The possibility that the presence of HHV-6 may prolong or intensify the GBS-related immune response should be considered in future studies since this cohort shows more severe neurological symptomatology in this group of patients.

It is important to note that the assay employed in this study did not differentiate between HHV-6a and HHV-6b; however, the literature suggests that both variants may be implicated in significant neurological diseases [34]. Furthermore, given their 95% genomic similarity, both viruses can infect a wide array of cells via the CD46 receptor, which is expressed in all nucleated human cells [35]. The spectrum of tissues susceptible to infection includes the brain, salivary glands, tonsils, liver, kidneys, lymph nodes, endothelial cells, and various leukocyte populations [14]. Notably, HHV-6A more readily infects neuronal cells and establishes lytic infections with cytopathic effects compared to HHV-6B [36].

Our study has some limitations. The small number of CSF samples is related to the reduced frequency of GBS during this period. Furthermore, as this is an exploratory study, we did not include samples from a control group due to the invasiveness and pain associated with the lumbar puncture procedure. qPCR in CSF is the gold standard methodology for confirming that the infectious process is occurring in the CNS. The blood–brain barrier is extremely sophisticated, and it is not common for microorganisms to be present in the CSF. A healthy group would not have the presence of infectious pathogens. The presence of an actively replicating virus in the CSF indicates that the infection is directly affecting the CNS.

Our data show a high frequency of individuals positive for HHV-6, and it is necessary to better understand the role of herpesviruses in GBS individuals. Molecular techniques, as well as traditional clinical methods, can improve the accuracy of diagnosis. Although it is not yet possible to say whether herpesviruses are a trigger for GBS or directly participate in the progression of neuronal injury, by accurately identifying them, new treatment possibilities may emerge. Further investigations are needed to determine whether treating these patients to control viral replication can have an impact on reducing the inflammatory process and symptoms in these individuals. Such interventions may reduce the length of hospital stay and may be an important measure to improve outcomes in this group of patients.

Incorporating routine qPCR analysis of CSF in Guillain–Barré syndrome can enhance diagnostic and prognostic accuracy by detecting and quantifying viral genetic material, such as herpesviruses. This method may help identify patient subgroups that could benefit from targeted antiviral treatments [37]. Although the pathophysiology of GBS differs from that of other neurological diseases, evidence from studies in multiple sclerosis supports the potential benefits of integrating molecular analyses into routine practice [38]. However, further research is necessary to validate this approach and develop specific clinical protocols.

Although it is not yet possible to say whether herpesviruses are a trigger for GBS or whether they influence its progression, by identifying herpesviruses in an accurate diagnosis, it is possible to treat these patients and control viral replication. This makes it possible to prevent GBS symptoms from being aggravated by the inflammatory process caused by the viruses.

## 5. Conclusions

In conclusion, this study provides a very comprehensive description with valuable information to improve the prognosis of GBS patients. Our findings indicate that HHV-6 may exacerbate the severity of Guillain–Barré syndrome, as evidenced by elevated viral loads in the cerebrospinal fluid. The results enhance the understanding of GBS prognosis and highlight the importance of monitoring herpesviruses in severe cases that may lead to prolonged hospitalization.

Furthermore, future studies could use these data to monitor severe GBS progression, management, and treatment outcomes.

## Figures and Tables

**Table 1:** Main data of GBS patients.

Patient	HHV-6	Co-Infection			Symptoms		LCR			Muscle Strength (MRC)				Motor Deficit	Electroneuromyography (Subtype)
			Sex	Age	First Day	Encephalitis	Cell Count (Cells/mm^3^)	Total Protein (mg/dL)	Glucose Level (mg/dL)	MMSS Proximal	MMSS Distal	MMII Proximal	MMII Distal		
1	Detected	yes	F	43	hypotension/paresis MMSS and MMII/muscle pain	no	1	124	70	3	3	2	2	TP	AIDP
2	Detected	yes	M	26	MMII paresis/MMII paresthesia	no	2	92	50	4	4	4	3	TP	AMSAN
3	Detected	yes	M	68	sensory paraplegia	somnolence	8	57	49	-	-	-	-	TP	AMSAN
4	Detected	yes	M	69	MMII paresis/MMII paresthesia	no	4	99	67	5	5	3	1	TP	AIDP
5	Detected	yes	M	83	paresis MMSS	no	1	24	37	2	3	4	4	TP	AIDP
6	Not detected	-	F	72	paresis MMSS and MMII/paraesthesia MMSS e MMII/pain in the hip and lower back	no	2	107	96	4	4	4	3	TP	AIDP
7	Not detected	no	M	58	paresis MMSS/paresthesia MMSS/pain in the face of the thigh	dysarthria	12	134	61	3	3	2	1	TP	AIDP
8	Not detected	-	M	39	paresthesia MMSS e MMII	no	1	33	80	4	3	4	3	paresis MMII	AMSAN
9	Not detected	no	F	50	paresthesia MMSS	no	1	104	58	4	4	3	4	TP	AIDP
10	Not detected	-	M	64	paresthesia MMSS e MMII	no	1	42	59	-	-	-	-	TP	AMSAN
11	Not detected	no	F	38	paresthesia MMSS	no	1	132	59	-	-	-	-	TP	AIDP
12	Not detected	-	F	49	paresthesiaMMSS	no	2	30	61	5	5	5	5	no	AIDP
13	Not detected	-	M	32	paresthesia MMII	no	1	91	-	4	4	4	4	TP	AIDP
Median positive patients HHV-6		-	-	68	-	-	2	92	50	3.5	3.5	3.5	2.5	-	-
Median negative patients HHV-6		-	-	49.5	-	-	1	97.5	61	4	4	4	3.5	-	-

MMSS: upper limbs; MMII: lower limbs; TP: progressive tetraparesis; AIDP: acute inflammatory demyelinating polyradiculoneuropathy; AMSAN: acute motor sensory axonal neuropathy.

**Table 2:** Characteristics of muscle strength and cerebrospinal fluid (CSF) cytochemical and biochemical findings obtained from participants at the time of admission.

Characteristics	Positive HHV-6			Negative HHV-6			*p*-Value
	Median	Min	Max	Median	Min	Max	
**CSF findings**							
Cell count (cells/mm^3^)	2	1	8	1	1	12	0.423
Total protein (mg/dL)	92	24	124	98	30	134	0.724
Glucose level (mg/dL) *	50	37	70	61	58	96	0.222
Muscle strength (MRC) **							
Proximal upper limbs	3.5	2	5	4	3	5	0.492
Distal upper limbs	3.5	3	5	4	3	5	0.820
Proximal lower limbs	3.5	2	4	4	2	5	0.492
Distal lower limbs	2.5	1	4	3.5	1	5	0.324

Min: minimum; max: maximum. * There is no information on a patient without HHV-6. ** There is no information on one patient with HHV-6 and two patients without HHV-6. Significance of the Mann–Whitney test: *p*-value < 0.05. MRC, Medical Research Council muscle strength scale.

**Table 3:** Frequency of herpesviruses and viral load.

Patient	HSV-1	HSV-2	VZV	EBV	HCMV	HHV-6	HHV-7	HHV-8
1	ND	ND	ND	ND	Detected	Detected	ND	ND
2	ND	ND	ND	ND	ND	Detected	ND	Detected
3	ND	ND	ND	ND	Detected	Detected	ND	ND
4	ND	Detected	ND	ND	Detected	Detected	ND	ND
5	ND	ND	Detected	ND	ND	Detected	ND	ND
6	ND	ND	ND	ND	ND	ND	ND	ND
7	ND	Detected	ND	ND	ND	ND	ND	ND
8	ND	ND	ND	ND	ND	ND	ND	ND
9	Detected	ND	ND	Detected	ND	ND	ND	ND
10	ND	ND	ND	ND	ND	ND	ND	ND
11	ND	Detected	ND	ND	ND	ND	ND	ND
12	ND	ND	ND	ND	ND	ND	ND	ND
13	ND	ND	ND	ND	ND	ND	ND	ND
Median CV	2.14 × 10^4^	4.51 × 10^4^	2.49 × 10^9^	1.63 × 10^5^	6.41 × 10^12^	1.50 × 10^12^	-	1.88 × 10^11^
Minimum CV	2.14 × 10^4^	4.15 × 10^4^	2.49 × 10^9^	1.63 × 10^5^	9.66 × 10^11^	8.24 × 10^8^	-	1.88 × 10^11^
Maximum CV	2.14 × 10^4^	4.78 × 10^4^	2.49 × 10^9^	1.63 × 10^5^	1.90 × 10^13^	2.00 × 10^13^	-	1.88 × 10^11^
Median CT	34.878	33.810	18.169	31.971	6.915	8.996	-	11.972
Minimum CT	34.878	33.725	18.169	31.971	5.360	5.282	-	11.972
Maximum CT	34.878	33.930	18.169	31.971	9.627	19.750		11.972

ND: not detected; CV: viral load (copies/mL); CT: cycle threshold (number of cycles required for amplification product.

## Data Availability

All data collected from patients are available in the tables throughout the article.

## Abbreviations

AD	Alzheimer’s disease
AFP	Acute flaccid paralysis
BBB	Blood–brain barrier
CNS	Central nervous system
CSF	Cerebrospinal fluid
EBV	Epstein–Barr virus
GBS	Guillain–Barré syndrome
HCMV	Human cytomegalovirus
HHV-6	Human herpesvirus 6
HHV-7	Human herpesvirus 7
HHV-8	Human herpesvirus 8
HSV-1	Herpes simplex virus 1
HSV-2	Herpes simplex virus 2
IVIG	Intravenous immunoglobulin therapy
MS	Multiple sclerosis
NPEVs	Non-polio enteroviruses
qPCR	Real-time PCR
VZV	Varicella-zoster virus

## References

[1] Sharma V., Chhabra T., Singh T.G. (2023). Correlation of COVID-19 and Guillain-Barré syndrome: A Mechanistic Perspective. Obes. Med..

[2] Shastri A., Al Aiyan A., Kishore U., Farrugia M.E. (2023). Immune-Mediated Neuropathies: Pathophysiology and Management. Int. J. Mol. Sci..

[3] Rodríguez Y., Rojas M., Pacheco Y., Acosta-Ampudia Y., Ramírez-Santana C., Monsalve D.M., Gershwin M.E., Anaya J.-M. (2018). Guillain–Barré syndrome, transverse myelitis and infectious diseases. Cell Mol. Immunol..

[4] Khanmohammadi S., Malekpour M., Jabbari P., Rezaei N. (2021). Genetic basis of Guillain-Barre syndrome. J. Neuroimmunol..

[5] Shahrizaila N., Lehmann H.C., Kuwabara S. (2021). Guillain-Barré syndrome. Lancet.

[6] Shahrizaila N., Yuki N. (2011). Guillain-Barré Syndrome Animal Model: The First Proof of Molecular Mimicry in Human Autoimmune Disorder. Biomed. Res. Int..

[7] Rojas M., Restrepo-Jiménez P., Monsalve D.M., Pacheco Y., Acosta-Ampudia Y., Ramírez-Santana C., Leung P.S.C., Ansari A.A., Gershwin M.E., Anaya J.-M. (2018). Molecular mimicry and autoimmunity. J. Autoimmun..

[8] Tam C.C., O’Brien S.J., Petersen I., Islam A., Hayward A., Rodrigues L.C. (2007). Guillain-Barré Syndrome and Preceding Infection with Campylobacter, Influenza and Epstein-Barr Virus in the General Practice Research Database. PLoS ONE.

[9] Vatti A., Monsalve D.M., Pacheco Y., Chang C., Anaya J.-M., Gershwin M.E. (2017). Original antigenic sin: A comprehensive review. J. Autoimmun..

[10] Eliassen E., Hemond C.C., Santoro J.D. (2020). HHV-6-Associated Neurological Disease in Children: Epidemiologic, Clinical, Diagnostic, and Treatment Considerations. Pediatr. Neurol..

[11] Carneiro V.C.d.S., Pereira J.G., de Paula V.S. (2022). Family Herpesviridae and neuroinfections: Current status and research in progress. Mem. Inst. Oswaldo Cruz.

[12] Pereira J.G., Leon L.A.A., de Almeida N.A.A., Raposo-Vedovi J.V., Fontes-Dantas F.L., Farinhas J.G.D., Pereira V.C.S.R., Alves-Leon S.V., de Paula V.S. (2023). Higher frequency of Human herpesvirus-6 (HHV-6) viral DNA simultaneously with low frequency of Epstein-Barr virus (EBV) viral DNA in a cohort of multiple sclerosis patients from Rio de Janeiro, Brazil. Mult. Scler. Relat. Disord..

[13] Miyazaki Y., Namba H., Torigoe S., Watanabe M., Yamashita N., Ogawa H., Morishima T., Yamada M. (2017). Monitoring of human herpesviruses-6 and -7 DNA in saliva samples during the acute and convalescent phases of exanthem subitum. J. Med. Virol..

[14] Agut H., Bonnafous P., Gautheret-Dejean A. (2015). Laboratory and Clinical Aspects of Human Herpesvirus 6 Infections. Clin. Microbiol. Rev..

[15] Hogestyn J., Mock D., Mayer-Proschel M. (2018). Contributions of neurotropic human herpesviruses herpes simplex virus 1 and human herpesvirus 6 to neurodegenerative disease pathology. Neural Regen. Res..

[16] Fokke C., van den Berg B., Drenthen J., Walgaard C., van Doorn P.A., Jacobs B.C. (2014). Diagnosis of Guillain-Barre syndrome and validation of Brighton criteria. Brain.

[17] Asbury A.K., Cornblath D.R. (1990). Assessment of current diagnostic criteria for Guillain-Barré syndrome. Ann. Neurol..

[18] Hati A., Chakraborty U., Chandra A., Biswas P. (2022). Hypokalaemia with Guillain-Barré syndrome: A diagnostic and therapeutic challenge. BMJ Case Rep..

[19] Carneiro V.C.d.S., Alves-Leon S.V., Sarmento D.J.d.S., Coelho W.L.d.C.N.P., Moreira O.d.C., Salvio A.L., Ramos C.H.F., Filho C.H.F.R., Marques C.A.B., Gonçalves J.P.D.C. (2022). Herpesvirus and neurological manifestations in patients with severe coronavirus disease. Virol. J..

[20] Sousa I.P., Burlandy F.M., Ferreira J.L., Alves J.C.S., Sousa-Júnior E.C., Tavares F.N., da Silva E.E. (2019). Re-emergence of a coxsackievirus A24 variant causing acute hemorrhagic conjunctivitis in Brazil from 2017 to 2018. Arch. Virol..

[21] Claverie J.-M., Santini S. (2021). Validation of predicted anonymous proteins simply using Fisher’s exact test. Bioinform. Adv..

[22] Mann H.B., Whitney D.R. (1947). On a Test of Whether one of Two Random Variables is Stochastically Larger than the Other. Ann. Math. Stat..

[23] Giorgi F.M., Ceraolo C., Mercatelli D. (2022). The R Language: An Engine for Bioinformatics and Data Science. Life.

[24] Awwad A., Ashmawi G., Hassan Y., Asser S. (2020). Association between Guillain-Barré syndrome and Herpes virus family members, A Multiplex PCR study. Egypt. J. Med. Microbiol..

[25] Stegmann-Planchard S., Gallian P., Tressières B., Leparc-Goffart I., Lannuzel A., Signaté A., Laouénan C., Cabié A., Hoen B. (2019). Chikungunya, a Risk Factor for Guillain-Barré Syndrome. Clin. Infect. Dis..

[26] Wijdicks E.F.M., Klein C.J. (2017). Guillain-Barré Syndrome. Mayo Clin. Proc..

[27] Dourado Junior M.E.T., Sousa B.F.d., Costa N.M.C.d., Jeronimo S.M.B. (2021). Cytomegalovirus infection in Guillain-Barré syndrome: A retrospective study in Brazil. Arq. Neuropsiquiatr..

[28] Anderson T.C., Leung J.W., Harpaz R., Dooling K.L. (2024). Risk of Guillain-Barré syndrome following herpes zoster, United States, 2010–2018. Hum. Vaccin. Immunother..

[29] Islam B., Islam Z., GeurtsvanKessel C.H., Jahan I., Endtz H.P., Mohammad Q.D., Jacobs B.C. (2018). Guillain-Barré syndrome following varicella-zoster virus infection. Eur. J. Clin. Microbiol. Infect. Dis..

[30] Brito C.A.A., Azevedo F., Cordeiro M.T., Marques E.T.A., Franca R.F.O. (2017). Central and peripheral nervous system involvement caused by Zika and chikungunya coinfection. PLoS Negl. Trop. Dis..

[31] Kim A.-Y., Lee H., Lee Y.-M., Kang H.-Y. (2021). Epidemiological Features and Economic Burden of Guillain-Barré Syndrome in South Korea: A Nationwide Population-Based Study. J. Clin. Neurol..

[32] van Leeuwen N., Lingsma H.F., Vanrolleghem A.M., Sturkenboom M.C.J.M., van Doorn P.A., Steyerberg E.W., Jacobs B.C. (2016). Hospital Admissions, Transfers and Costs of Guillain-Barré Syndrome. PLoS ONE.

[33] Shahan B., Choi E.Y., Nieves G. (2021). Cerebrospinal Fluid Analysis. Am. Fam. Physician.

[34] Komaroff A.L., Pellett P.E., Jacobson S. (2021). Human Herpesviruses 6A and 6B in Brain Diseases: Association versus Causation. Clin. Microbiol..

[35] Tang H., Serada S., Kawabata A., Ota M., Hayashi E., Naka T., Yamanishi K., Mori Y. (2013). CD134 is a cellular receptor specific for human herpesvirus-6B entry. Proc. Natl. Acad. Sci. USA.

[36] De Bolle L., Van Loon J., De Clercq E., Naesens L. (2005). Quantitative analysis of human herpesvirus 6 cell tropism. J. Med. Virol..

[37] Lycke J., Svennerholm B., Hjelmquist E., Frisén L., Badr G., Andersson M., Vahlne A., Andersen O. (1996). Acyclovir treatment of relapsing-remitting multiple sclerosis. J. Neurol..

[38] Bergström T. (1999). Herpesviruses—A rationale for antiviral treatment in multiple sclerosis. Antiviral Res..

